# Supplementary material for: Rare non-coding variants are associated with plasma lipid traits in a founder population
Source: Sci Rep. 2017 Nov 27;7:16415. doi: 10.1038/s41598-017-16550-8 (PMC5704019; doi:10.1038/s41598-017-16550-8)

Rare non-coding variants are associated with plasma lipid traits in a founder population

Catherine Igartua[1*], Sahar V Mozaffari[1,2], Dan L Nicolae[1,3,4], Carole Ober[1,2]

[1]Department of Human Genetics, University of Chicago, Chicago, IL, 60637
[2]Committee of Genetics, Genomics and Systems Biology, University of Chicago, Chicago, IL, 60637
[3]Department of Statistics, University of Chicago, Chicago, IL, 60637
[4]Department of Medicine, University of Chicago, Chicago, IL, 60637

*Corresponding Author: cigartua (at) uchicago.edu

Supplemental Data

Supplementary Figure 1: Quantile-quantile (QQ) plots for plasma lipid trait GWAS of rare in European variants. Dashed blue line represents the genome-wide significant threshold ($p<5x10^{-8}$).

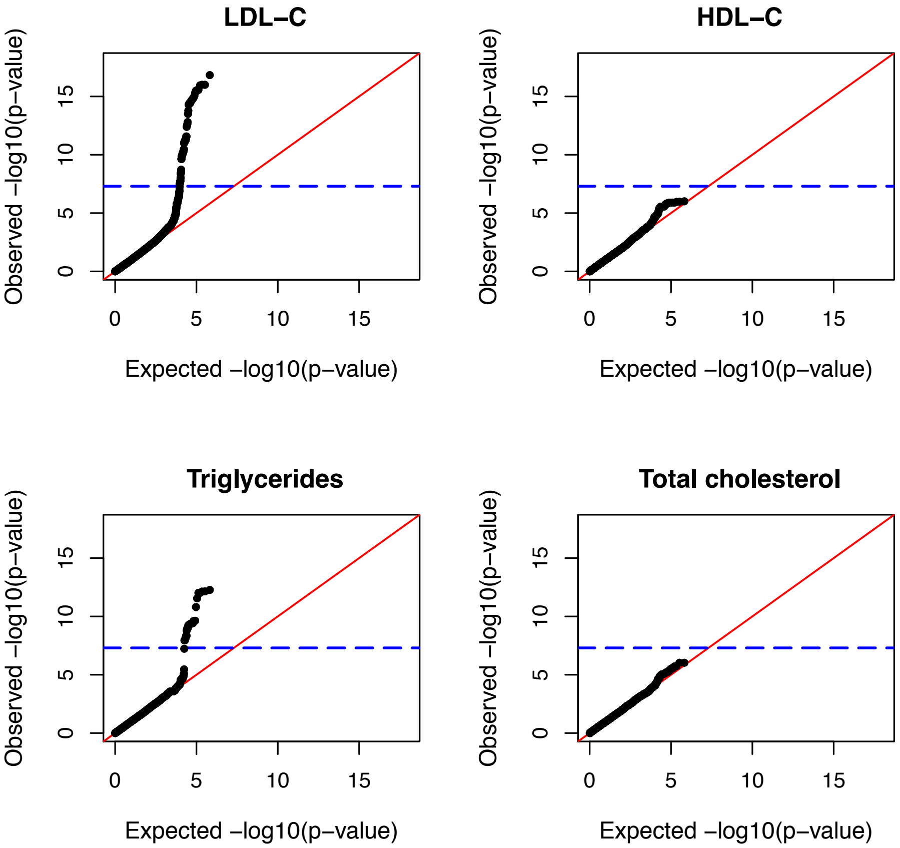

Supplementary Figure 2: Manhattan plots for plasma lipid trait GWAS of rare in European variants. Red line shows the genome-wide significant threshold ($p<5x10^{-8}$). Labeled variants are the most significant variant at each genome-wide significant locus.

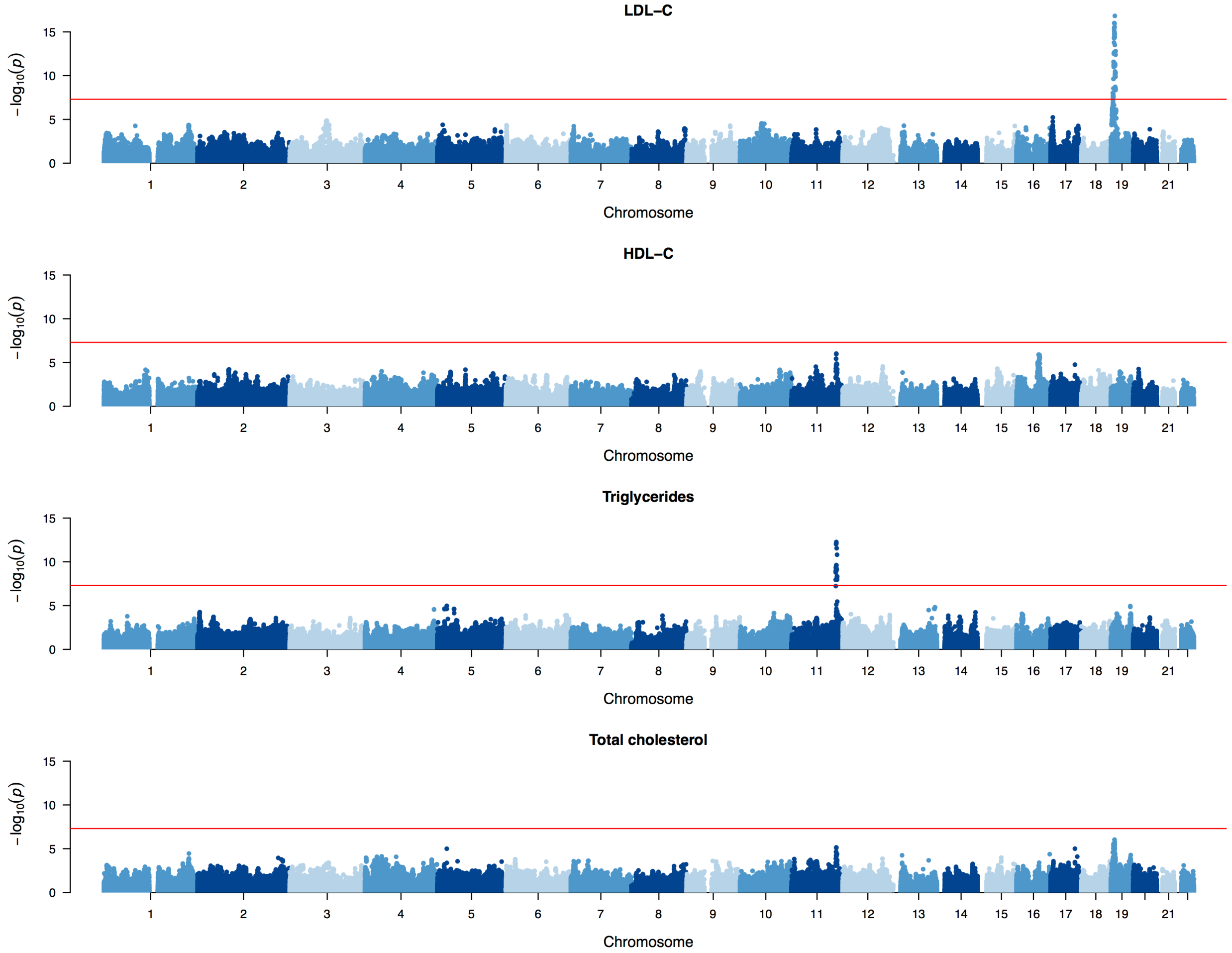

Supplementary Table 1: Rare variants in Europeans fgwas combined model for LDL-C. The parameters inferred from a single annotation model for LDL-C along and the marginal effect of each annotation in the final model are shown.

Parameter	Description	log_2(effect) [95% CI]	Penalized effect	Marginal effect [95% CI]
Gene-rich region	Regional annotation: top 1/3 of gene density	0.45 [0.13,0.74]	0.47	0.41 [0.07,0.70]
Gene-poor region	Regional annotation: bottom 1/3 of gene density	-5.27 [-20,-1.62]	-2.38	-25.37 [-40,-1.82]
Enhancer Primary Natural Killer cells from peripheral blood	Epigenome Roadmap putative enhancer region[a]	2.48 [1.10,3.63]	1.97	2.20 [0.70,3.26]
Enhancer H1 BMP4 Derived Trophoblast Cultured Cells	Epigenome Roadmap putative enhancer region[a]	1.26 [0.14,2.19]	1.08	1.33 [0.11,2.29]
Enhancer Stomach Mucosa	Epigenome Roadmap putative enhancer region[a]	-2.52 [-5.49,-0.87]	-1.52	-0.43 [-3.41,1.16]
H3K4me1_Trynka	H3K4me1 peaks post-processed by Trynka *et al.* (2013)[b]	0.83 [0.11,1.67]	0.85	1.15 [0.37,2.04]

[a]Putative enhancer region delineated using DNAse I regions (-log(pvalue)>10), annotated with the 5 mark-15 state model on observed data across 127 epigenomes (ROADMAP + ENCODE). Data downloaded from Reg2Map Honeybadger2. [b]Bed files downloaded from data published in Finucane *et al.* (2015)[60].

Supplementary Table 2: Rare variants in Europeans fgwas combined model for TG levels. Presented are the parameters inferred from a single annotation model for TG levels and the marginal effect of each annotation in the final model.

Parameter	Description	log_2(effect) [95% CI]	Penalized effect	Marginal effect [95% CI]
Gene-rich region	Regional annotation: top 1/3 of gene density	0.21 [-0.62,0.86]	0.11	0.09 [-0.75,0.73]
Gene-poor region	Regional annotation: bottom 1/3 of gene density	0.07 [-0.84,0.74]	-0.02	-0.02 [-0.93,0.66]
Transcr_Hoffman	Transcribed regions defined as the union over the six cell lines from the combined chromHMM and Segway annotations in Hoffman *et al.* (2013) [b]	-1.59 [-3.63,-0.01]	-1.50	-1.99 [-4,-0.42]
Enhancer Breast Myoepithelial Primary Cells	Epigenome Roadmap putative enhancer region[a]	29.66 [0.86,49.66]	1.21	1.48 [-0.28,3.02]
Noncoding transcript variant	A transcript variant of a noncoding RNA gene	-0.52 [-2.61,1.06]	-0.48	-0.58 [-2.72,1.04]
Enhancer_Hoffman extend 500	Enhancer regions extended by 500bp defined as the union over the six cell lines from the combined chromHMM and Segway annotations in Hoffman et al. (2013) [b]	-28.17 [-48.17,0.12]	-1.43	-7.79 [-20,-0.28]
FetalDHS_Trynka extend 500	Fetal DNAse hypersensitivity site extended by 500bp. Combination of ENCODE and Roadmap Epigenomics DHS data, post-processed by Trynka et al. (2013)[b]	-0.82 [-2.16,0.29]	-0.66	-0.77 [-2.06,0.33]
Enhancer Breast variant Human Mammary Epithelial Cells (vHMEC)	Epigenome Roadmap putative enhancer region[a]	1.25 [-1.19,2.74]	1.10	1.57 [-0.65,3.76]
DGF_ENCODE	Digital genomic footprinting (DGF) from Encode[b]	-0.07 [-1.90,1.19]	-0.29	-0.41 [-2.03,0.82]
Enhancer_Andersson extend 500	FANTOM5 enhancers extended by 500bp were obtained from Andersson *et al.* (2014)[b]	-0.05 [-20,2.171]	0.02	0.12 [-20,2.15]

[a]Putative enhancer region delineated using DNAse I regions (-log(pvalue)>10), annotated with the 5 mark-15 state model on observed data across 127 epigenomes (ROADMAP + ENCODE). Data downloaded from Reg2Map Honeybadger2. [b]Bed files downloaded from data published in Finucane *et al.* (2015)[60].

Supplementary Table 3: Rare variants in Europeans fgwas combined model for HDL-C. Presented are the parameters inferred from a single annotation model HDL-C and the marginal effect of each annotation in the final model.

Parameter	Description	log_2(effect) [95% CI]	Penalized effect	Marginal effect [95% CI]
Enhancer iPS DF 19.11 Cells	Epigenome Roadmap putative enhancer region[a]	34.14 [2.27,54.14]	2.69	4.42 [0.75,20]
Enhancer Fetal Intestine Small	Epigenome Roadmap putative enhancer region[a]	-159.72 [-179.72,-2.23]	-1.37	-28.77 [-48.77,1.86]
Promoter Breast variant Human Mammary Epithelial Cells (vHMEC)	Epigenome Roadmap putative enhancer region[a]	68.72 [-0.57,88.71]	0.60	33.60 [1.93,53.6]

[a]Putative enhancer region delineated using DNAse I regions (-log(pvalue)>10), annotated with the 5 mark-15 state model on observed data across 127 epigenomes (ROADMAP + ENCODE).

Supplementary Table 4: Rare variants in Europeans fgwas combined model for total cholesterol. Presented are the parameters inferred from a single annotation model for total cholesterol and the marginal effect of each annotation in the final model.

Parameter	Description	$\log_2$(effect) [95% CI]	Penalized effect	Marginal effect [95% CI]
Enhancer iPS DF 19.11 Cells	Epigenome Roadmap putative enhancer region[a]	36.91 [1.66,56.91]	2.84	33.79 [3.19,53.79]
Enhancer_Hoffman extend 500	Enhancer regions extended by 500bp defined as the union over the six cell lines from the combined chromHMM and Segway annotations in Hoffman et al. (2013)[b]	5.83 [-20,20]	-1.11	-85.52 [-105.52,-1.12]

[a]Putative enhancer region delineated using DNAse I regions (-log(pvalue)>10), annotated with the 5 mark-15 state model on observed data across 127 epigenomes (ROADMAP + ENCODE). Data downloaded from Reg2Map Honeybadger2. [b]Bed files downloaded from data published in Finucane *et al.* (2015)[60].

Supplementary Figure 3: Rare variants on chromosomes 1 and 3 are suggestively associated with increased LDL-C. (**a, c**). *Locus plots.* The top panel shows the p-values of association with LDL-C for all variants discovered in the Hutterites regardless of allele frequency in Europeans. Symbols correspond to the maximum allele frequency in Europeans, with closed circles representing REVs (see legend), and are colored based on their LD r^2 with the most associated variant in the region (rs191020975 and chr1:224811120, respectively). The next three panels show tracks for the annotations selected in the fgwas joint model, annotations of known GWAS loci from NHGRI and genes in the region. **(b, d).** *Genotype boxplots for LDL-C for candidate intronic variants CNIH3 (chr1:224811120; p=7.99x10^{-5}) and in EPHA6 (rs191020975; p=1.88Ex10^{-5}).*

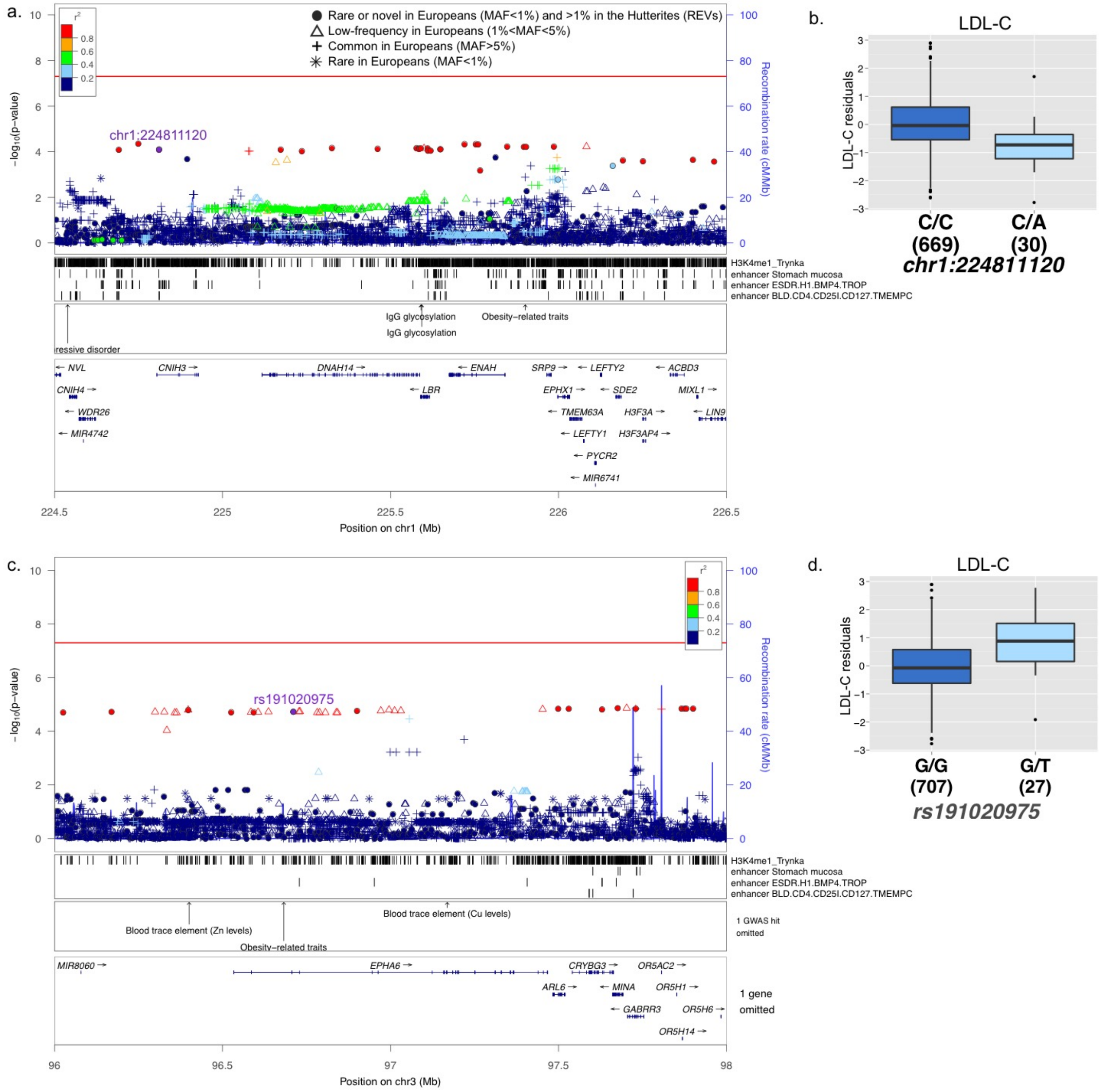

Supplementary Figure 4: Rare variants on chromosome 11 are associated with HDL-C. (*a*) *Locus plots.* The top panel shows the p-values of association with HDL-C for all variants discovered in the Hutterites regardless of allele frequency in Europeans. Symbols correspond to the maximum allele frequency in Europeans, with closed circles representing REVs (see legend), and are colored based on their LD r^2 with the most associated variant in the region (rs184333869). The next three panels show tracks for the annotations selected in the fgwas joint model, annotations of known GWAS loci from NHGRI and genes in the region. **(b)** Genotype boxplots for rs184333869 *QTL associated with HDL-C levels* (*TMPRSS4-AS1; $p=1.10x10^{-6}$).*

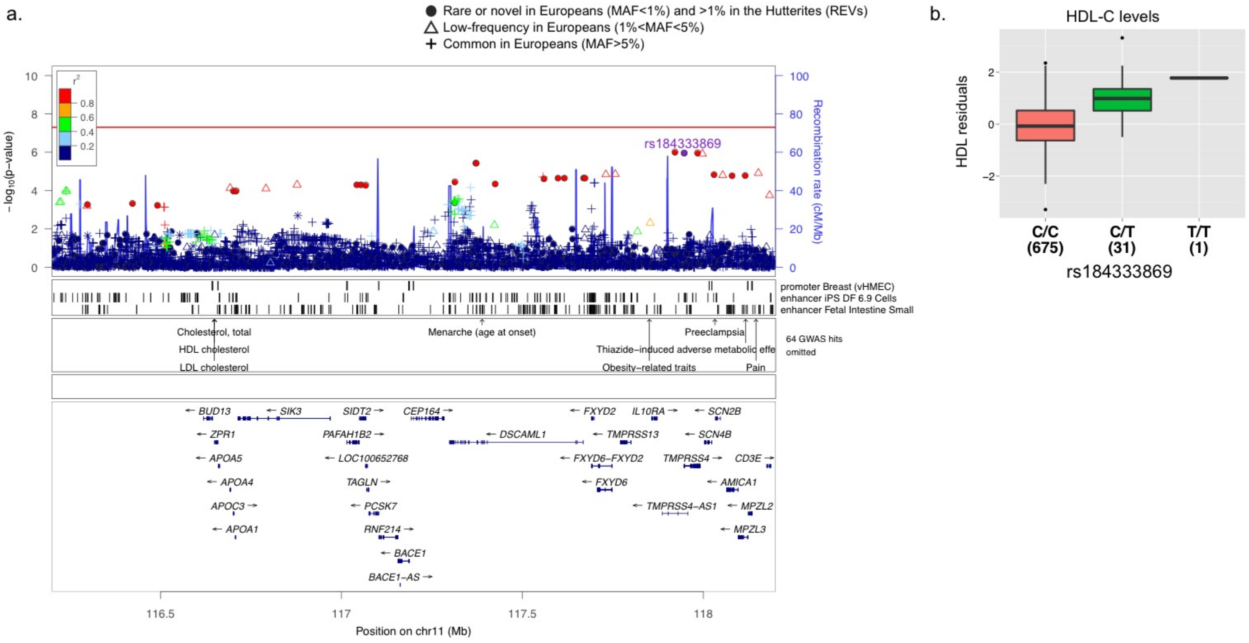

Supplementary Figure 5: *APOC3* and *ZPR1* QTL haplotype boxplots for triglyceride levels. Alleles were phased for the lead common signal identified in conditional analysis (rs11604424; top) and the candidate *APOC3* rare splicing variant (rs13826449; bottom). The trends observed between each of five haplotype combinations present in our sample show these variants exert opposite effects on TG levels.

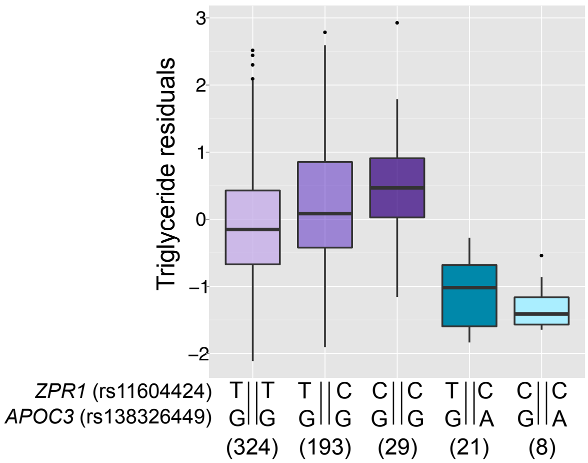

Supplement: Supplementary file 1 — Supplementary information [file 41598_2017_16550_MOESM1_ESM.pdf]
